# Metabolic engineering for the production of isoprene and isopentenol by *Escherichia coli*

**DOI:** 10.1007/s00253-018-9200-5

**Published:** 2018-07-14

**Authors:** Meijie Li, Rui Nian, Mo Xian, Haibo Zhang

**Affiliations:** 1 Key Laboratory of Biobased Materials, Qingdao Institute of Bioenergy and Bioprocess Technology, Chinese Academy of Sciences, No. 135 Songling Road, Qingdao, 266101 People’s Republic of China; 2 University of Chinese Academy of Sciences, Beijing, 100049 People’s Republic of China

**Keywords:** Isoprene, Isopentenol, MVA, MEP, Metabolic engineering

## Abstract

The biotechnological production of isoprene and isopentenol has recently been studied. Isoprene, which is currently made mainly from petroleum, is an important platform chemical for synthesizing pesticides, medicines, oil additives, fragrances, and more and is especially important in the rubber production industry. Isopentenols, which have better combustion properties than well-known biofuels (ethanol), have recently received more attention. Supplies of petroleum, the conventional source of isoprene and isopentenols, are unsustainable, and chemical synthesis processes could cause serious environmental problems. As an alternative, the biosynthesis of isoprene and isopentenols in cell factories is more sustainable and environmentally friendly. With a number of advantages over other microorganisms, *Escherichia coli* is considered to be a powerful workhorse organism for producing these compounds. This review will highlight the recent advances in metabolic engineering for isoprene and isopentenol production, especially using *E. coli* cell factories.

## Introduction

Isoprenoids comprise a large variety of natural products varying in structure and function, many of which are important chemicals, such as flavors, fragrances, pigments, antioxidants, steroids, and drugs (Sacchettini and Poulter 1997). Isoprenoids are classified according to their number of five-carbon units, which are their basic building blocks (Ruzicka 1953). Hemiterpenoids consist of one unit and include isoprene, isopentenols (prenol and isoprenol), and other chemicals. All isoprenoids are derived from the universal C_5_ prenyl phosphate precursors isopentenyl diphosphate (IPP) and dimethylallyl diphosphate (DMAPP), which are supplied by the methylerythritol phosphate (MEP) pathway or the mevalonate (MVA) pathway. Then, downstream steps utilize IPP and DMAPP to produce a number of isoprenoids (Klein-Marcuschamer et al. 2007).

In the synthetic chemistry industry, isoprene (C_5_H_8_) can be used as a platform chemical for a variety of products, such as rubbers, adhesives, elastomers, pesticides, medicines, oil additives, fragrances, and biofuels (Claeys et al. 2004; Ruzicka 1953). Approximately 95% of isoprene is used for rubber synthesis, and approximately 20 million tons of rubber is produced annually (Yang et al. 2012b). Currently, isoprene is produced industrially from cracking petroleum, an unrecoverable and environment-unfriendly resource (Whited et al. 2010). Isoprene collection from plants is another production method. Isoprene is naturally produced by many plants and is emitted into the earth’s atmosphere in teragram quantities (Grawert et al. 2004; Sharkey et al. 2008; Sharkey and Yeh 2001). However, its volatility makes this collection difficult. Moreover, plant cultivation is seasonally dependent and requires land resources, which hinders large-scale production (Gupta and Phulara 2015; Vickers et al. 2014). There is a major need for sustainable industrial isoprene production methods. Metabolic engineering of microbial systems for isoprene production might be a feasible alternative to address these problems. The well-studied microbial host *Escherichia coli* has been mostly used for the bio-production of isoprene with heterologous MVA pathway introduction or endogenous MEP pathway modification and isoprene synthase (IspS) introduction (Klein-Marcuschamer et al. 2007; Martin et al. 2003).

Isopentenols, including isoprenol and prenol, currently obtained from petroleum-based isobutene and formaldehyde, have recently received attention as advanced biofuels. These biofuels show some attractive characteristics, such as higher energy density, higher octane numbers, lower water miscibility, and better low-temperature fluidity than other biofuels (Gupta and Phulara 2015). These combustion properties make isopentenols a potential substitute for petroleum. Notably, isopentenols have significant knock resistance and can improve the anti-knock properties of petroleum as additives (Mack et al. 2014). As isoprenoids, isopentenols can also be obtained from the common precursors IPP and DMAPP, which are dephosphorylated by phosphatase. Similar to isoprene, metabolic engineering of cell factories to improve isopentenol production has been applied.

Furthermore, the metabolic engineering of cell factories for isoprene and isopentenol production is also instructive and meaningful for engineering the bio-production of other isoprenoids. To some extent, the MEP and MVA pathways are shared by all isoprenoids, and metabolic engineering methods for the biosynthesis of isoprenoids are mutually referential. However, metabolic engineering of the pathway into naive microbial hosts can result in the accumulation of IPP and DMAPP, which are toxic for bacterial growth (George et al. 2018). Therefore, balancing the accumulation and consumption of IPP and DMAPP is very important for isoprenoid bio-production (Klein-Marcuschamer et al. 2007). Unlike other isoprenoids, isoprene and isopentenol are direct derivatives of IPP and DMAPP, which makes controlling the accumulation of IPP and DMAPP easier than other isoprenoids. Therefore, engineering methods for isoprene and isopentenol production are likely easier than engineering methods for other isoprenoids, although they are still generally instructive, especially for methods related to the balance of the IPP/DMAPP pool.

Bio-isoprene production and related research progress have been previously summarized in several reviews. The material and energy efficiencies, cost and economic evaluation of chemical- and biological-based isoprene production were estimated and compared by Morais et al. in 2015 (Morais et al. 2015). Isoprene production methods using petrochemical and biological sources were first described by Genencor in 2010, in a summary of their patents (Whited et al. 2010). Engineering of isoprene production in microbes was simply described as a case study in a review on isoprenoid production in 2014 and 2015 (Vickers et al. 2014; Whited et al. 2010; Ye et al. 2016). In the latest review, bio-isoprene production in different cell factories was reviewed (Ye et al. 2016). Thirteen articles on bio-isoprene production have been published in the last 2 years, representing almost half of the total published studies concerning isoprene bio-production since 2001. Novel engineering approaches have been applied and great improvements in isoprene production have been achieved, especially in the *E. coli* cell factories, which are considered as a powerful workhorse organism for producing a wide range of biofuels and chemicals and have garnered more attention. In addition, the bio-production of isopentenol has not been reviewed.

In this review, we summarize the biotechnological production of isoprene and isopentenol, especially in *E. coli* cell factories. Five sections are divided according to the target of the engineering approach: IspS for isoprene production, phosphatase for isopentenol production, the natural MEP pathway, the heterogeneous MVA pathway, and precursor support. The key enzymes and applied engineering approaches in *E. coli* are described. Challenges and prospects for future engineering are also discussed in this review.

## Use of IspS for isoprene production

Even though the MEP pathway is naturally expressed in *E. coli*, no IspS has been identified. To improve isoprene production in *E. coli*, the introduction of IspS is required. In plants, IspS has been proven to be the limiting enzyme in the MEP pathway, which is related to the effect of isoprene release in plants, as it has a protective effect against environmental stresses (Sharkey et al. 2008). Isoprene is an aeriform product, unlike most other organic products, and feedback inhibition is avoided through this pathway. These qualities make IspS the bottleneck for isoprene production. Moreover, enzyme assays have indicated that most studied IspS enzymes have low activity and low affinity for the substrate DMAPP, as indicated by low *k*_cat_ values and high *K*_m_ values in the millimolar range (Schnitzler et al. 2005). IspS is essential for the industrial-scale production of isoprene. Research on IspS has focused mainly on improving the activity and efficiency of the enzyme, including screening new IspS enzymes and performing directed evolution and other metabolic engineering strategies.

### Screening of IspS

IspS enzymes from several organisms have been identified and utilized for isoprene bio-production in *E. coli*. In an early-stage study, IspS from *Populus* spp., such as white poplar, black poplar, and gray poplar, were identified, and white poplar led to the highest isoprene production in *E. coli* (Table 1) (Yang et al. 2012b; Zhao et al. 2011). Moreover, IspS from *Pueraria montana* (kudzu) also has been widely utilized. In different studies, the measured *k*_cat_ values of IspS from kudzu were different, and variable isoprene production was obtained (Table 1). In a recent study, more IspSs from poplar were identified, and an approximately 3-fold isoprene production increase was detected (Table 1) (Kim et al. 2016). Novel IspS enzymes from other plants (*Ipomoea batatas*, *Mangifera indica*, *Elaeocarpus photiniifolius*, and *Mucuna pruriens*) have been identified through genome mining, and high isoprene production has been detected (Table 1) (Ilmen et al. 2015). IspS enzymes from *Eucalyptus globulus* even have a 5-fold isoprene production increase over the strain engineered with IspS from white poplar (Table 1) (Gao et al. 2016; Hayashi et al. 2013). Correspondingly, IspS from *E. globulus* has a higher *k*_cat_ value than IspS from white poplar (Table 1).

**Table 1:** Summary of the IspS enzymes utilized for isoprene production

IspS source	Enzyme activity^a^	Isoprene production^b^	Number of studies	Reference
*P. alba*	*K*_m_ = 8.7/15.9 mM *k*_cat_ = 0.03/0.034 s^−1^	1	12	Oku et al. 2015; Sasaki et al. 2005
*P. montana* (kudzu)	*K*_m_ = 7.7/2.5 mM *k*_cat_ = 0.088/4.4 s^−1^	2.3/0.3/0.25	9	Ilmen et al. 2015; Kim et al. 2016; Sharkey et al. 2005; Yang et al. 2012b; Zurbriggen et al. 2012
*P. nigra*	–	0.8	1	Yang et al. 2012b
*E. globulus*	*K*_m_ = 0.2 mM *k*_cat_ = 0.195 s^−1^	5	1	Gao et al. 2016
*I. batatas*	–	1.5	1	Ilmen et al. 2015
*P. trichocarpa*	–	3	1	Kim et al. 2016

^a^Different *K*_m_ and *K*_cat_ values for IspS from *P. alba* and *P. montana* were obtained in different studies

^b^Isoprene production of the strain engineered with *P. alba* was considered to be 1, and isoprene production of the strains engineered with IspS from other organisms were compared to this value

IspS enzymes have been identified in several plants since the first IspS was cloned from gray poplar in 2001. Moreover, while isoprene has been successfully detected in microbes, such as *Bacillus subtilis*, no IspS-encoding gene has yet been identified in microorganisms (Julsing et al. 2007; Sivy et al. 2002). IspS enzymes in different species show similarities in characteristic indexes. Most IspS enzymes show optimal enzyme activity at temperatures of 40–45 °C, with some exceptions at 35 and 50 °C (Lehning et al. 1999; Schnitzler et al. 1996). IspS from plants has an optimum pH between 7 and 10, preferring alkaline conditions, similar to other enzymes with botanical origins (Oku et al. 2015). IspS shows relatively high *K*_m_ values for DMAPP (in the millimolar range) and low *k*_cat_ values (in the range of 0.011–1.7 s^−1^) (Table 1), which indicate that high concentrations of DMAPP are required and low levels of isoprene are produced. Of note, the temperature and pH preferences of IspS are not consistent with *E. coli* culture conditions. During fermentation, IspS shows low enzyme activity, and the metabolic flux for isoprene production is seriously hampered at this point. For bio-isoprene production, especially in *E. coli*, screening IspS enzymes for appropriate biochemical properties for expression in microbes is still a promising approach.

### Directed evolution of IspS

**Fig. 1:**
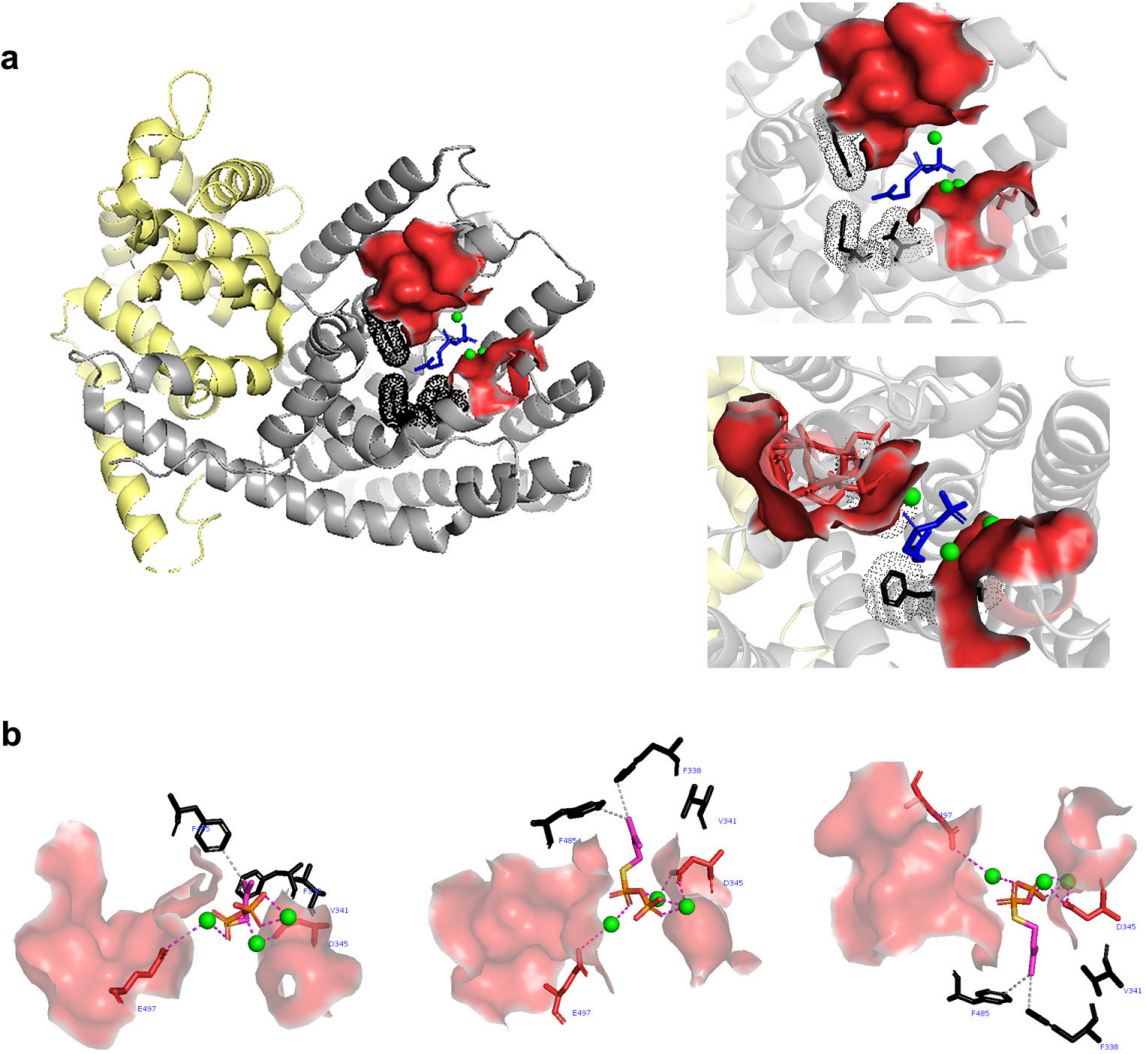
Structural information of IspS from gray poplar with the substrate DMAPP and the co-factor Mg^2+^. **a** Information of the two identified active motifs and special residues. The N-terminal domain (yellow) and the active domain (gray) are displayed. The conserved DDXXD motif (red) and the NSE/DTE motif (red) are located in the active C-terminal domain. The substrate DMAPP (blue) and three Mg^2+^ co-factors (green) are located between the motifs. The three special residues (dark), F338, V341, and F485, are located just outside of the active pocket and supposedly reduce the size of the substrate-binding site. **b** Interaction of particular residues with DMAPP and Mg^2+^. Three different views are shown. The interactions between DMAPP and residues are indicated with gray dashed lines. The interactions between Mg^2+^ and DMAPP or residues are indicated with purple dashed lines. The PDB number of the IspS from gray poplar is 3N0G

**Fig. 2:**
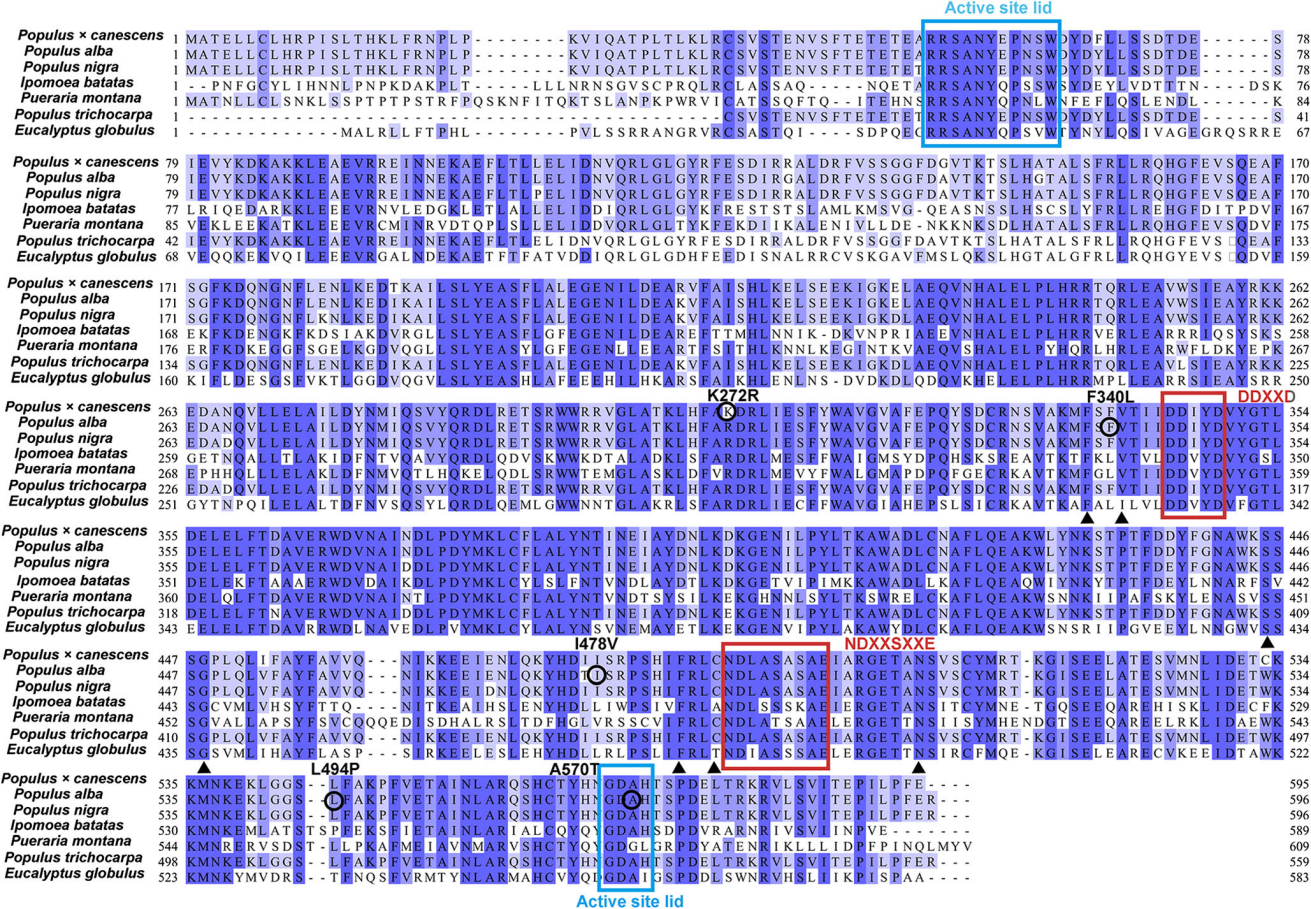
Sequence alignment of IspSs from several organisms. The blue and red rectangle indicates the two identified active site lid motifs and the two conserved metal-binding motifs. Seven residues that have been identified as having special enzyme activities are indicated with small black triangles. The amino acid mutations applied in research studies are also indicated. The accession numbers of the IspSs listed are CAC35696.1, AB198180.1, ADV58934.1, MH084470, MH084471, ACD70404.1, and BAF02831.1

Although information on the three-dimensional structure of IspS is currently not very clear, the structure of IspS from gray poplar (*Populus × canescens*) was identified (Fig. 1). The IspS enzyme includes an N-terminal α-barrel class II terpenoid synthase fold and an active C-terminal α-helical class I terpenoid synthase fold (Fig. 1a). In the active domain, two metal-binding motifs were identified, a DDXXD conserved motif and an NSE/DTE conserved motif (Fig. 1a and Fig. 2). D345 in the DDXXD motif and E497 in the NSE/DTE motif supposedly interact with the metal ion (Fig. 1a) (Koksal et al. 2010; Sharkey et al. 2005, 2013). In addition, several amino acids related to enzyme activity were identified. Some amino acids in white poplar, gray poplar, and kudzu were suggested as the substrate-binding site (Fig. 1b and Fig. 2) (Koksal et al. 2010; Sharkey et al. 2005, 2013). Among them, the two phenylalanine (F) residues are unique to IspS and supposedly reduce the size of the substrate-binding site to ensure that larger substrates, such as GPP and FPP, will not fit in the binding site (Fig. 1a) (Ilmen et al. 2015).

Based on the information provided by the three-dimensional structure, several amino acid mutations were applied, resulting in increased enzyme activity (Fig. 2). In white poplar, mutation of amino acid L494 led to an approximately 2-fold increase in enzyme activity (Beck et al. 2013a). Interestingly, IspSs from other organisms commonly contain residue P in the homologous site (Fig. 2). For gray poplar, the K272R mutation shows a 1.8- to 2-fold increase in activity relative to wild type (Bott et al. 2014). Except for IspS from gray poplar, an R residue is located in the same position (Fig. 2). Three positive mutations, F340L, I478V, and A570T, were identified in white poplar, and mutated amino acids revealed essential roles in enzyme activity (Wang et al. 2017). In the homologous F340 site, amino acid L is adopted in IspSs from three other species, *I. batatas*, *P. montana*, and *E. globulus*, and the utilization of these IspSs resulted in improved bio-isoprene production (Table 1 and Fig. 2). Similarly, residue V present in the homologous I478 site in IspSs was speculated to have better enzyme activity (Table 1 and Fig. 2). Mutation information on IspS is not only helpful for understanding the enzyme structure but is also instructive for enzyme engineering of IspSs for bio-isoprene production in microbes. In a recent study, directed evolution of IspS from white poplar was performed, and a 3-fold increase in isoprene production was obtained (Wang et al. 2017).

### Other metabolic engineering strategies for IspSs in *E. coli*

For isoprene production in *E. coli*, a plant-derived IspS is heterogeneously expressed, and metabolic engineering methods are applied to improve enzyme expression and activity. First, codon optimization and removal of the chloroplast-targeting peptide were utilized (Lindberg et al. 2010; Yang et al. 2012b). A 1-fold increase in isoprene production was achieved by deleting the 5′ part of the *IspS* sequence from gray poplar (Miller et al. 2001). Finding the appropriate expression vector was another essential process. For *IspS* from kudzu, among the tested vectors, the low copy vector, pCL1920, produced more isoprene than other vectors (Cervin et al. 2012). Improving enzyme activity is another powerful strategy. In recent studies, as a rate-limiting enzyme with high *K*_m_ value, IspS was fused with IDI to improve the interaction between the substrate DMAPP and IspS (Bentley and Melis 2012; Bentley et al. 2014; Lindberg et al. 2010). In another study, interestingly, the introduction of monoterpene or sesquiterpene synthase, which consumes excessive DMAPP, into an engineered strain resulted in increased isoprene production (Chotani et al. 2013).

However, even though IspSs from different plant species have been utilized, and several engineering methods have been applied in the last 15 years, the production of isoprene from DMAPP is still seriously hampered. Directed evolution of IspS is still a challenge, and high-throughput screening is difficult, as isoprene is a volatile gas, and the substrate, DMAPP, is not easy to monitor. A screening method based on the cytotoxicity of DMAPP was constructed (Wang et al. 2017). In the near future, identifying IspSs from other organisms with better activity and establishing more stable high-throughput screening methods may offer powerful breakthroughs.

## Use of phosphatase for isopentenol production

**Fig. 3:**
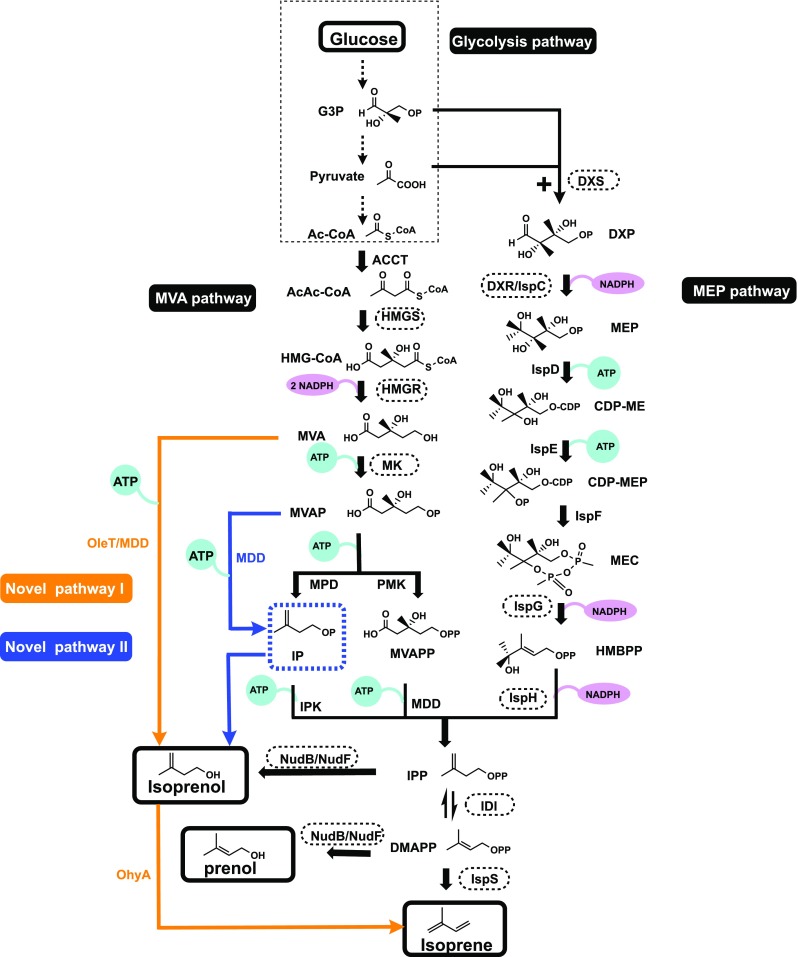
The metabolic pathways for isoprene and isopentenol production. The precursors of the pathway are produced through the glycolysis pathway. Enzymes circled by dashed lines indicate the rate-limiting points. NADPH and ATP consumption are indicated. The orange and blue lines indicate the novel pathways, respectively. Ac-CoA, acetyl-CoA; AcAc-CoA, acetoacetyl-CoA; HMG-CoA, 3-hydroxy-3-methylglutaryl-CoA; MVA, mevalonate; MVAP, mevalonate-5-phosphate; MVAPP, mevalonate-5-pyrophosphate; IP, isopentenyl phosphate; G3P, glyceraldehyde-3-phosphate; DXP, 1-deoxy-d-xylulose-5-phosphate; MEP, methylerythritol phosphate; CDP-ME, 4-diphosphocytidyl-2C-methyl-d-erythritol; CDP-MEP, 4-diphosphocytidyl-2C-methyl-d-erythritol-2-phosphate; MEC, 2C-methyl-d-erythritol-2,4-cyclo-diphosphate; HMBPP, 4-hydroxy-3-methyl-2-(E)-butenyl-4-diphosphate; IPP, isopentenyl diphosphate; DMAPP, dimethylallyl diphosphate; ACCT, acetoacetyl-CoA thiolase; HMGS, HMG-CoA synthase; HMGR, HMG-CoA reductase; MK, mevalonate kinase; PMK, MVAP kinase; MDD, MVAPP decarboxylase; MPD, MVAP decarboxylase; IPK, IP kinase; DXS, DXP synthase; DXR/IspC, DXP reductoisomerase; IspD, CDP-ME cytidylyltransferase; IspE, CDP-ME kinase; IspF, MEC synthase; IspG, HMBPP synthase; IspH, HMBPP reductase; IDI, isopentenyl-diphosphate isomerase; IspS, isoprene synthase; MDD, MVAPP decarboxylase; OleT, fatty acid decarboxylase; NudB/NudF, ADP-ribose pyrophosphatase

Isopentenols, including isoprenol and prenol, show attractive combustion properties, and the bio-production of isopentenol in *E. coli* has been studied in the last 10 years. Dephosphorylation of IPP and DAMPP by an appropriate phosphatase leads to the formation of isopentenol (Fig. 3). To improve isopentenol production, the identification of a powerful phosphatase is key. The enzymes YhfR and NudF from *B. subtilis* were primarily identified to catalyze this dephosphorylation process after screening of a 19,000 clone library by monitoring the cytotoxicity of IPP and DMAPP (Withers et al. 2007). Isopentenols were successfully detected when *BsNudF* was introduced into engineered *E. coli* (Withers et al. 2007). Similarly, a homogeneous enzyme, EcNudB, from *E. coli* was reported to have a phosphatase effect after screening of the HAD and Nudix superfamilies (Chou and Keasling 2012). The identified enzymes show different affinities for IPP and DAMPP. EcNudB exhibited a high substrate preference for DMAPP, and BsNudF showed equivalent affinity for IPP and DAMPP, with the same amounts of isoprenol and prenol detected (Zheng et al. 2013). However, the identified phosphatases both show low affinity for IPP and DMAPP. Correlation analysis indicated IPP accumulation in the engineered *E. coli* (Zheng et al. 2013). This IPP accumulation can be slightly relieved by increased EcNudB expression. Furthermore, a fusion protein of IDI and EcNudB increased the metabolic flux from IPP and DMAPP to isopentenol, resulting in increased prenol production and decreased isoprenol production (Chou and Keasling 2012).

## Modification of the natural MEP pathway in *E. coli*

The MEP pathway exists in *E. coli* naturally, and this pathway has been modified. Engineered *E. coli* in which only IspS was introduced showed low levels of isoprene production (Miller et al. 2001). Promoting flux through core isoprenoid pathways has been shown to be the most powerful way to increase isoprene and isopentenol production in *E. coli*.

### Description of the MEP pathway

Rohmer et al. demonstrated the existence of the MEP pathway in 1993, breaking the conventional concept that the MVA pathway is the only pathway to synthesize DMAPP and IPP (Rohmer et al. 1993). The enzymes and biochemistry of each step of the MEP pathway have been analyzed since that time (Fig. 3) (Grawert et al. 2011; Hunter 2007). Converting the central carbon intermediates pyruvate and glyceraldehyde-3-phosphate (G3P) to DMAPP or IPP requires eight reactions catalyzed by nine enzymes (Fig. 3). 1-Deoxy-d-xylulose-5-phosphate (DXP) is produced by DXP synthase (DXS), which catalyzes the condensation of pyruvate and G3P. DXP is converted into MEP by DXP reductoisomerase (DXR or IspC). MEP is transformed into 2C-methyl-d-erythritol-2,4-cyclo-diphosphate (MEC) by three sequential steps in which two intermediate products, 4-diphosphocytidyl-2C-methyl-d-erythritol (CDP-ME) and 4-diphosphocytidyl-2C-methyl-d-erythritol-2-phosphate (CDP-MEP), are produced. These three steps are catalyzed by CDP-ME cytidylyltransferase (IspD), CDP-ME kinase (IspE), and MEC synthase (IspF). MEC is converted to 4-hydroxy-3-methyl-2-(E)-butenyl-4-diphosphate (HMBPP) by HMBPP synthase (IspG). The production of IPP is catalyzed by HMBPP reductase (IspH). Isopentenyl-diphosphate isomerase (IDI) is responsible for the conversion between DMAPP and IPP.

Even though the process of the MEP pathway has been elucidated, the utilization of this pathway in *E. coli* has not resulted in high efficiency. All of the enzymes except IspS are natural enzymes; therefore, the problems in heterogeneous introduction would not be faced in the production of isoprene with an engineered MEP pathway in *E. coli*. Regulatory mechanisms at the transcriptional and translational levels should bring more attention to further research, which will have a significant impact on the microbial production of isoprenoids.

### Restrictive points of the MEP pathway

To maximize the production of IPP and DMAPP for isoprene synthesis, a complete understanding of the regulatory mechanisms of the metabolic flux through the MEP pathway is of tremendous importance. It is proposed that the MEP pathway may have several potential control points, DXS, DXR, IspD, IspF, IspG, IspH, and IDI, with different enzymes exhibiting different degrees of control (Fig. 3). DXS has been shown to be a key rate-limiting enzyme in microbes and plants (Lois et al. 2000; Rodriguez-Concepcion and Boronat 2002). It is also feedback-regulated by the intermediates IPP and DMAPP, which bind with DXS and lead to its inactivation (Banerjee et al. 2013). DXR appears to be another rate-limiting enzyme (Rodríguez-Concepción 2006). A positive correlation between increased isoprenoid accumulation and the overexpression of DXR was found in peppermint (Mahmoud and Croteau 2001). The last two steps of the MEP pathway, catalyzed by IspG and IspH, are also considered to be regulatory nodes (Rodríguez-Concepción 2006). IspG and IspH contain a dioxygen-sensitive iron-sulfur [4Fe–4S] cluster that participates in the reduction process with the presence of a redox shuttle, such as flavodoxin/flavodoxin reductase/NADPH (Seemann et al. 2002; Wolff et al. 2003). Overexpression of IspG prevents the efflux of MEC, resulting in an increase of downstream isoprenoid production in *E. coli* (Zhou et al. 2012). In some species, the rate-limiting role of IspG parallels the release of upstream bottlenecks. Without the overexpression of DXS, increased carotenoid accumulation was not achieved by only the IspG up-regulation in *E. coli* (Flores-Perez et al. 2008). IDI has been proven to be another rate-limiting enzyme in isoprenoid production. A 1.4-fold increase in β-carotene production was achieved by introducing a strong promoter for IDI expression (Yuan et al. 2006). In a few studies, IspD and IspF were also rate-limiting enzymes. Combinational overexpression of IspD and IspF by replacing the wide promoter with a strong promoter led to increased β-carotene production (Yuan et al. 2006). However, IspD and IspF appear to be weaker bottlenecks, and they are often ignored in the metabolic engineering of isoprenoid production in common research. In engineered *E. coli*, DXS, DXR, and IDI are usually overexpressed to promote MEP pathway flux, with a resulting positive effect.

### Metabolic engineering of the MEP pathway in *E. coli*

With the existence of several bottlenecks, the MEP pathway has been engineered to improve isoprene production. As strong bottlenecks, DXS and DXR are mostly engineered to promote metabolic flux. The classic engineering methods, overexpression of the native genes and introduction of genes from other organisms, are performed. In an engineered *E. coli* strain, recruitment of DXS and DXR from *B. subtilis* led to a 314 mg/L isoprene yield, which was 2-fold higher than the strain with native DXS and DXR overexpression (Zhao et al. 2011). Furthermore, to improve enzyme activity, directed co-evolution of DXS/DXR/IDI was applied, and improvement in isoprene production by 60% was achieved (Lv et al. 2016). Two other restrictive points, IspG and IspH, are also focused to further improve the metabolic flux. In an engineered *E. coli* strain, in which DXS, IDI, and IspS were overexpressed, MEC, the substrate of IspG, was observed to accumulate, and the metabolic flux was hindered. Three further approaches were applied to solve this problem. First, *gcpE* (encoding IspG) and *lytB* (encoding IspH) from *Thermosynechococcus elongatus* were introduced into *E. coli*, resulting in increased isoprene production (Chotani et al. 2013). Second, IscR, which encodes an enzyme responsible for repressing the expression of iron–sulfur [4Fe–4S]-containing genes, was deleted from the host (Akhtar and Jones 2008; Chotani et al. 2013). Third, the catalytic activity of IspG and IspH can be improved by flavodoxin and flavodoxin-NADP^+^ oxidoreductase. Overexpression of the reduction shuttle-related genes and endogenous *fldA* and introduction of *PetF* and *PetH* from *T. elongatus* led to improved enzyme activity and promoted metabolic flux in *E. coli* (Chotani et al. 2013). The MEP pathway has also been employed for isopentenol production by overexpressing endogenous IspG and DXS and exogenous expression of YhfR and NudF from *B. subtilis* in *E. coli*, with a total of 61.9 mg/L titer obtained by 5-L-scale batch cultivation (Liu et al. 2014).

For isoprene and isopentenol production, most researchers focus on the rate-limiting enzymes and perform common engineering methods, such as overexpression, direct evolution, and gene deletion, as described above, to improve enzyme expression or enzyme activity. As the MEP pathway was discovered only 20 years ago, the regulatory mechanisms and enzymes of this metabolic pathway are poorly studied. A better understanding of the MEP pathway is necessary, and further optimization strategies for isoprene and isopentenol production in *E. coli* need to be explored.

## Introduction of the heterogeneous MVA pathway in *E. coli*

### Description of the MVA pathway

The MVA pathway is an alternative pathway that is also utilized for isoprene and isopentenol production. The MVA pathway naturally operates in the cytoplasm of most eukaryotes, fungi, and plants, while the MEP pathway functions in many bacteria, algae, cyanobacteria, and plant chloroplasts. However, even though the MEP pathway is naturally operational in *E. coli*, research utilizing the MEP pathway for the production of isoprene or other isoprenoids is far less advanced than research introducing the whole MVA pathway. The MVA pathway was first discovered in the 1950s, 40 years earlier than the MEP pathway, and it has been better elucidated than the MEP pathway. The regulatory mechanisms and biochemistry of the MVA pathway are well characterized. Utilization of the MVA pathway for the industrial production of isoprenoids has been extensively performed, and great success has been obtained. Briefly, the MVA pathway is divided into two pathways, the upper pathway and the lower pathway (Fig. 3). The upper pathway includes three steps from acetyl-CoA to MVA, which are catalyzed by acetoacetyl-CoA thiolase (ACCT), 3-hydroxy-3-methylglutaryl-CoA (HMG-CoA) synthase (HMGS), and HMG-CoA reductase (HMGR) (Fig. 3). MVA is converted into IPP through the lower pathway via three reactions (Fig. 3). Mevalanote-5-phosphate (MVAP) is produced from MVA by mevalonate kinase (MK). MVAP is transformed into IPP through two different pathways. In eukaryotes, IPP is produced from MVAP through phosphorylation and decarboxylation steps, which are catalyzed by MVAP kinase (PMK) and MVAPP decarboxylase (MDD), respectively. However, in archaea, the decarboxylation occurs first, and MVAP is converted to IPP by two enzymes, MVAP decarboxylase (MPD) and isopentenyl phosphate (IP) kinase (IPK). IPP is subsequently converted into DMAPP through an isomerization reaction catalyzed by IDI. The structural and functional basis for every enzyme has been described in detail and was summarized by Miziorko (2011).

### Restrictive points of the MVA pathway

Like the MEP pathway, metabolic flux through the MVA pathway is tightly regulated by several control nodes (Fig. 3). In the upper pathway, HMGR is proposed to be a key enzyme controlling the carbon flux in different species. In mammals, HMGR is a rate-limiting enzyme and the primary target of cholesterol-lowering drug therapy for sterol and cholesterol synthesis through MVA pathway (Burg and Espenshade 2011). In plants, an improvement in isoprenoid production has been detected after the overexpression of HMGR (Stermer et al. 1994). These studies indicate that HMGR plays an essential role in the metabolic flux through the MVA pathway, and optimization of this point is necessary. In contrast to HMGR, ACCT and HMGS appear to subtly regulate the carbon flux. Similarly, the lower MVA pathway is tightly hampered by several restrictive points. The first step, catalyzed by MK, is strongly influenced by feedback regulation at transcriptional and post-translational levels (Hinson et al. 1997). Even through MK is not naturally expressed in *E. coli*, it is tightly regulated by the metabolites of downstream pathways, namely, DMAPP, IPP, GPP, and FPP, as well as other isoprenoids in bacteria and eukaryotes. In another study, targeted proteomics analysis has also identified two potential bottlenecks, MK and MPK, in engineered *E. coli* (Redding-Johanson et al. 2011). IDI is identified as another rate-limiting point through the MVA pathway and has been described in the MEP pathway section. Even though the upper pathway is regulated by three points, when heterogeneously expressed in *E. coli*, the metabolic flux restriction can be solved by appropriate modification (described below). However, the restrictive points in the lower pathway are not easy to eliminate. Mevalonate accumulation is detected in most engineered *E. coli* hosts. Moreover, with IspS showing high *K*_m_ and low *k*_cat_, IPP and DMAPP accumulate, regulating the expression of upstream genes in a feedback manner. The metabolic flux limitation in the lower MVA pathway is serious, and research on further regulation mechanisms is necessary.

### Metabolic engineering of the MVA pathway in *E. coli*

As a pathway that does not naturally exist in *E. coli*, introduction of the whole pathway from other organisms and modification of the restrictive points are necessary for bio-isoprene and isopentenol production. MVA pathways from different organism sources have been implemented in *E. coli* through the approaches of genome integration or the introduction of independent plasmid constructs. MVA pathways from different organisms exhibit different enzyme efficiencies. A strain utilizing the upper pathway from *Enterococcus faecalis* showed a 5-fold increase in isoprene production compared with a strain utilizing the upper pathway from *Saccharomyces cerevisiae* (Yang et al. 2012a, b). Genome integration is a powerful approach to enhance stable gene expression in *E. coli*. In cyanobacteria, integration of the upper pathway from *E. faecalis*, lower pathway from *Streptococcus pneumoniae*, an additional *AtoB* from *E. coli*, and an *IspS* from kudzu into the chromosome have been applied for isoprene production (Bentley et al. 2014). This approach has not been applied in *E. coli*, and further improvement may result. As an alternative to whole pathway engineering, optimization of the rate-limiting enzymes (HMGS and MK) through the MVA pathway can also significantly improve isoprene production. Studies have indicated that the enzymatic reaction increased dramatically (140-fold) after alanine 110 of HMGS from *E. faecalis* (mvaS) was mutated to glycine; corresponding isoprene production increased 2.3-fold (Steussy et al. 2006; Yang et al. 2012a). Selection of MK from *Methanosarcina mazei*, a feedback-resistant enzyme, is often applied for isoprenoid production. A 5-fold increase in isoprene accumulation was achieved when an additional MK from *M. mazei* was expressed in *E. coli* (Beck et al. 2014). Furthermore, heterogeneous expression of the MVA pathway in *E. coli* is enhanced by choosing appropriate translation initiation regions (TIRs). In the engineered *E. coli* strain, the introduction of TIRs from *E. coli* upstream of each gene in the lower pathway from *S. pneumoniae* resulted in a 5-fold increase in isoprene production compared to the utilization of *S. pneumoniae* native TIRs (Zurbriggen et al. 2012).

Though engineering methods of the MVA pathway in *E. coli* have been implemented, metabolic flux of the pathway is still seriously limited, especially in the lower pathway. Accumulation of MVA is detected in most engineered *E. coli* hosts. Several approaches have been applied to circumvent this problem. To improve the consumption of MVA, a novel pathway that circumvents the rate-limiting steps has been applied (Yang et al. 2016b). In this novel pathway, MVA is catalyzed by fatty acid decarboxylase (OleT_JE_) to isoprenol, which is catalyzed by oleate hydratase (OhyA_EM_) to produce isoprene (Fig. 3). Although the productivity of this novel pathway is very low, screening for enzymes with better affinity and activity may be a promising solution. MDD, which shows promiscuous decarboxylase activity toward MVA and MVAP, was utilized for isoprenol production in another study (Kang et al. 2015). In novel pathway I, isoprenol is produced from MVA directly, catalyzed by MDD. In novel pathway II, MVAP is catalyzed by MDD to produce IP, which is dephosphorylated by phosphatase to produce isoprenol. Both novel pathways circumvent the toxic accumulation of IPP and DMAPP and show decreased ATP consumption. However, novel pathway II showed more isoprenol production than novel pathway I. In fact, novel pathway II is similar to the MVA pathway in archaea, in which MVAP is catalyzed by MPD to produce IP, which is phosphorylated by IPK to produce IPP (Fig. 3). Therefore, novel pathway II shows more potential for isoprenol production, and screening powerful enzymes capable of catalyzing MVAP to IP will be necessary in the future. As an alternative to cell factories, cell-free systems utilizing the MVA pathway have also been attempted. Our group has established a cell-free system in which five enzymes were utilized to produce isoprene from mevalonate, and the optimized enzyme ratio was analyzed, preventing the accumulation of intermediates (Cheng et al. 2017). In the last 2 years, several studies have been performed attempting to assess bio-isoprene production in other microorganisms with the introduction of the MVA pathway and *IspS*. Isoprene production has been successfully detected in engineered *S. cerevisiae*, *Bacillus* spp., cyanobacteria, and *Clostridium ljungdahlii* (Chaves et al. 2017; Diner et al. 2018; Gomaa et al. 2017; Wang et al. 2017). However, until now, the highest isoprene production was still obtained from engineered *E. coli* with the whole MVA pathway and IspS engineered. Utilization of these approaches, novel pathways, cell-free system, and other cell factories are still in the exploratory stage and need further research.

As an alternative to utilization of the MVA or MEP pathway alone, synergy between the two pathways was performed, resulting in approximately 20-fold and 3-fold increases relative to strains with only an engineered MEP or MVA pathway, respectively (Yang et al. 2016a). In this strain, the restrictive points of the MEP pathway and the whole MVA pathway were engineered. However, the communication between MEP and MVA pathway is complicated, and engineering of both of the pathways separately in the same strain is not sufficient. In recent research, when only the MVA pathway was introduced into an *E. coli* host, the intermediate product of the MEP pathway, MEC, accumulated substantially, which indicated that the native MEP pathway flux was seriously influenced when the MVA pathway was introduced (George et al. 2018). More information on the connection between these two pathways may be deciphered in the near future and will be helpful for isoprene production engineering of *E. coli* hosts.

Although the MEP and MVA pathways have been widely applied for isoprene production, large differences in production have resulted. Strains utilizing the native MEP pathway have lower isoprene production than strains engineered by introducing the heterogeneous MVA pathway. Theoretically, both pathways have their respective pros and cons, as reviewed by Cao et al. (2018). The MEP pathway shows a higher theoretical yield, and the MVA pathway is more energetically friendly. It is speculated that the NAD(P)H and ATP consumption levels of the MEP pathway are a heavy burden for isoprene production. Furthermore, the native MEP pathway is strictly regulated by the cell system. The regulatory mechanism in *E. coli* may counteract the flux promotion via the overexpression of key points, DXS and DXR. In contrast, the exogenous MVA pathway acts more aggressively, without the corresponding regulation. Of course, accumulation of intermediates of the MVA pathway is also detected, which explains the decreased cell density when the MVA pathway is introduced.

## Optimization of precursor support

A large number of studies are highly focused on optimization of the MEP or the MVA pathway to improve isoprene production. However, sufficient support of the precursors, G3P and pyruvate in the MEP pathway and acetyl-CoA in the MVA pathway, also plays essential roles.

**Fig. 4:**
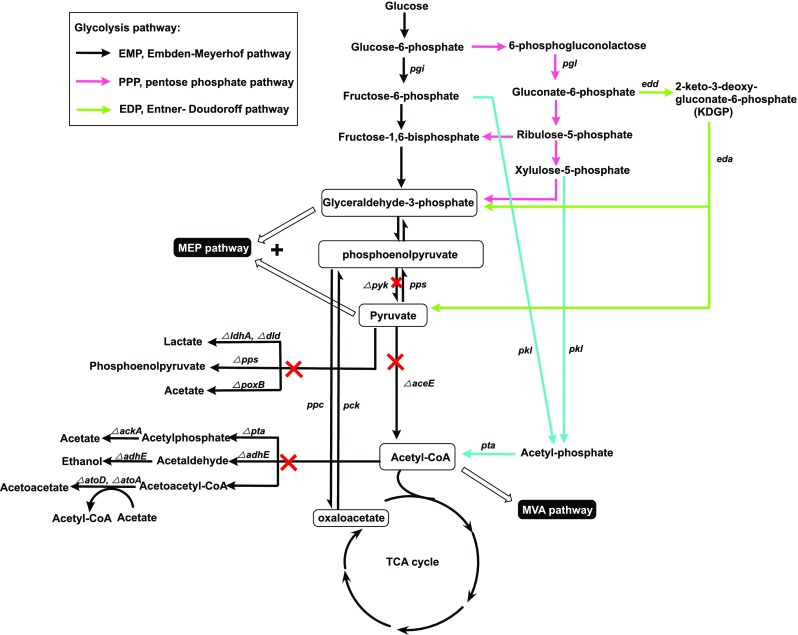
Metabolic engineering of the glycolysis pathway in *E. coli* for precursor support of the MEP and MVA pathways. *pgi*, phosphoglucose isomerase; *pgl*, 6-phosphogluconolactonase; *adhE*, aldehyde-alcohol dehydrogenase; *dld*, d-lactate dehydrogenase; *eda*, KDGP-aldolase; *edd*, 6-phosphogluconate dehydrase; *ackA*, acetate kinase; *pkl*, phosphoketolase; *pta*, phosphotransacetylase; *pps*, phosphoenolpyruvate (PEP) synthase; *ppc*, PEP carboxylase; *pck*, PEP carboxykinase; *atoA*, acetoacetyl-CoA transferase; *pyk*, pyruvate kinases; *aceE*, pyruvate dehydrogenase; *ldhA*, lactate dehydrogenase; *poxB*, pyruvate oxidase; *pta*, phosphate acetyltransferase; *atoD*, acetoacetyl-CoA transferase

For the MEP pathway, a sufficient supply of the precursors G3P and pyruvate is essential for high-level production of isoprene and many other commercially interesting isoprenoids. When glucose is used as a carbon source, four glycolysis pathways were tested, and a combination of the EDP and PPP pathways, which generate G3P and pyruvate simultaneously, led to increase in isoprene and isopentenol production (Fig. 4) (Chotani et al. 2013; Liu et al. 2013, 2014). In *E. coli*, improved lycopene production resulted when *aceE*, which encodes a pyruvate dehydrogenase, was eliminated, redirecting the precursor pyruvate from the glycolysis pathway to the MEP pathway (Fig. 4) (Alper et al. 2005). An equitable balance of G3P and pyruvate is also necessary for metabolite flux to the MEP pathway. Overexpression of *pps* and *pck* and deletion of *pyk*, which redirects the flux from pyruvate back to G3P and leads to a balance between these components, improved lycopene production (Fig. 4) (Farmer and Liao 2001).

When the MVA pathway is utilized, marked improvement of the accumulation of the precursor acetyl-CoA is essential for high-level production. Phosphoketolase (pkl), which catalyzes the direct conversion of xylulose-5-phosphate or fructose-5-phosphate into acetyl-CoA without carbon loss, allows for the production of three acetyl-CoAs per glucose. Overexpression of pkl from *Enterococcus gallinarum* resulted in higher utilization efficiency of glucose and higher cumulative isoprene yield (Fig. 4) (Beck et al. 2015). Isoprene production can also be enhanced by overexpression of *pgl*, encoding a 6-phosphogluconolactonase, which improves flux through the PPP and suppresses the glycosylation of heterogeneous enzymes, providing enough acetyl-CoA for the MVA pathway (Fig. 4) (Beck et al. 2013b; Cervin et al. 2012). In *E. coli*, acetyl-CoA is involved in several pathways. Impeding the pathways for byproduct formation from precursor acetyl-CoA is a powerful method for improving isoprene production (Fig. 4). In an engineered *E. coli* strain, knockout genes related to byproducts such as lactate, acetate, and ethanol formation led to improved isoprene production, reaching 1832 mg/L, the highest among all of the publications (Fig. 4) (Kim et al. 2016). Precursors of the MEP and MVA pathways are involved in numerous essential pathways that are essential for organism growth. Overexpression or deletion of particular genes may have complicated effects on the engineered strain. Therefore, studies on the engineering of precursor support are scarce.

Considering production costs, appropriate support of carbon sources is very important for the production of isoprene and other isoprenoids in industry. Several carbon sources, such as corn stover, bagasse, softwood pulp, and glucose, have been tested, and softwood pulp produced the highest amount of isoprene, slightly less than the amount produced by glucose (Cervin et al. 2012). Isoprene production was also detected in the engineered strain, which consumed fatty acids or palm oil (Cervin et al. 2012). Conversion of glycerol to isoprene was achieved in *E. coli* by recruiting the MVA pathway and isoprene synthase through overexpression of glpK and glpD, which promotes glycerol dissimilation (Bredow et al. 2015). Glycerol was found to be superior to glucose as a carbon source for isoprenoid production. In one study, glycerol yielded the highest β-carotene production and cell growth among the various carbon sources tested (Yoon et al. 2009). In another study, using glycerol as the major carbon source and glucose and l-arabinose as auxiliary carbon sources improved lycopene production (Kim et al. 2011). When the carbon source was galactose, the De Ley–Doudoroff (DD) pathway was selected, which produces equivalent amounts of G3P and pyruvate (Ramos et al. 2014).

## Conclusion and prospects

Isoprene is an essential platform chemical in industrial applications, especially in rubber synthesis. Microbial isoprene production has been studied for the past 18 years to address the multiple negative influences of petroleum consumption, as petroleum is the original source of not only isoprene but also many other organic chemicals important to industry. *E. coli*, which is perhaps the most technically mature host for genetic manipulation and heterologous expression, is widely chosen for isoprene production to introduce the whole heterologous MVA pathway or modify the inherent MEP pathway and heterologously express isoprene synthetase from plants. A few limitation points of the metabolic flux have been identified, and optimization of the key enzymes with approaches such as protein engineering and large-scale screening has been implemented. However, even though high isoprene production has been achieved, there is still much room for improvement. Multiple steps are needed for isoprene production from its common precursors, and monitoring every step can help modulate enzyme expression, facilitating smooth carbon flux. In addition, multiple steps mean greater consumption of resources, and the exploration of novel metabolic pathways is a promising approach for isoprene production. In recent years, many other hosts aside from *E. coli* and *S. cerevisiae* have been well characterized and utilized for chemical production. Though other producers, such as cyanobacteria, *Bacillus* spp., and *Clostridium ljungdahlii*, have been engineered, *E. coli* still exhibits unique advantages, including isoprene yield, its technical maturity as a host for genetic manipulation, and heterologous expression.

Microbial isopentenol production has been applied in the last 10 years. The enzyme responsible for isopentenol production from IPP and DMAPP has been identified. However, improvements in isopentenol production have been restricted by the low activity of this enzyme. Screening powerful enzymes may be a feasible improvement strategy. Recently, inspiring results have been achieved by building a truncated MVA pathway, and further improvements in isopentenol productivity are promising.
